# Cell-Wall Hydrolases as Antimicrobials against Staphylococcus Species: Focus on Sle1

**DOI:** 10.3390/microorganisms7110559

**Published:** 2019-11-12

**Authors:** Aurore Vermassen, Régine Talon, Carine Andant, Christian Provot, Mickaël Desvaux, Sabine Leroy

**Affiliations:** 1 Université Clermont-Auvergne, INRA, MEDIS, F-63000 Clermont-Ferrand, France; aurore.vermassen@educagri.fr (A.V.); regine.talon@inra.fr (R.T.); carine.andant@inra.fr (C.A.); mickael.desvaux@inra.fr (M.D.); 2 BioFilm Control, Biopôle Clermont Limagne, F-63360 Saint-Beauzire, France; christian.provot@biofilmcontrol.com

**Keywords:** cell wall hydrolases, staphylococci, lytic activity, phylogeny

## Abstract

Some staphylococcal species are opportunistic pathogens of humans and/or animals with *Staphylococcus epidermidis* as one of the most important. It causes a broad spectrum of diseases in humans and animals. This species is able to form biofilms and has developed antibiotic resistance, which has motivated research on new antibacterial agents. Cell-wall hydrolases (CWHs) can constitute a potential alternative. Following a hijacking strategy, we inventoried the CWHs of *S. epidermidis*. The lytic potential of representative CWHs that could be turned against staphylococci was explored by turbidity assays which revealed that cell wall glycosidases were not efficient, while cell wall amidases and cell wall peptidases were able to lyse *S. epidermidis*. Sle1, which is encoded by chromosomal gene and composed of three anchoring LysM domains and a C-terminal CHAP (cysteine, histidine-dependent amidohydrolase/peptidase) domain, was one of the most active CWHs. The phylogeny of Sle1 revealed seven clusters mostly identified among staphylococci. Sle1 was able to lyse several staphylococcal species, including *Staphylococcus aureus*, both in planktonic and sessile forms, but not *Micrococcus*.

## 1. Introduction

Staphylococci are mainly associated with the skin, skin glands and mucous membranes of animals and humans [1,2]. The genus includes more than 51 species (www.bacterio.net) divided into two groups according the coagulase test, with nine species of coagulase positive staphylococci (CPS) and a vast group of coagulase negative species (CNS). Some species are opportunistic pathogens of humans and/or animals with *Staphylococcus aureus* (CPS) and *Staphylococcus epidermidis* (CNS) as the most important ones. They cause a broad spectrum of diseases ranging from skin diseases, wound sepsis, mastitis, endocarditis, osteomyelitis, and lung infections in humans and animals [2,3]. In a survey of the prevalence of healthcare-associated infections (HAI) and antimicrobial use in Europe, 7.1% of patients had at least one HAI and 34.6% received one antimicrobial agent (e.g., antibiotic) [4]. Staphylococci were commonly isolated from HAI and represented 35.2% of all types of infections, 54.3% at surgical sites and 41.7% in blood stream infections. *S. aureus* was found in 12.1% of all types of infections and in 21.5% of surgical site infections, with 34.2% of strains resistant to methicillin. CNS accounted for 8.3% of all types of infections, 13.4% of surgical site infections, and 16.7% of blood stream infections [4]. Among CNS species, *S. epidermidis* is predominant in clinical isolates and can act as a reservoir of antibiotic resistance genes for *S. aureus* [2,5]. The population structure of *S. epidermidis* appears epidemic with the emergence of well-adapted clones evolving rapidly through genetic recombination by frequent transfer of genetic mobile elements such as staphylococcal cassette chromosome mec (SCCmec) elements [2]. Furthermore, *S. epidermidis* as well as *S. aureus* are able to form biofilms on biotic and abiotic surfaces [3,6,7,8,9].

The antibiotic resistance of these two species has motivated research on new antibacterial agents. Cell-wall hydrolases (CWHs) constitute a potential alternative. These ubiquitous bacterial enzymes hydrolyze peptidoglycan and play a key role in cell growth, separation and turnover but also in cell lysis. They are classified into three classes according their conserved catalytic domains, amidases, glycosidases and peptidases [10]. Only a few CWHs are described in staphylococci, including lysostaphin, Atl, Sle1 and LytN [10,11]. Lysostaphin isolated from *Staphylococcus simulans* is one of the most studied cell-wall peptidases (CWPs). It cuts the peptide bond between the third and fourth glycine residues of the pentaglycine cross-link in the peptidoglycan of staphylococci [11,12]. The major hydrolase Atl (AtlA in *S. aureus*, AtlE in *S. epidermidis* and AtlL in *Staphylococcus lugdunensis*) is a bifunctional enzyme composed of one cell-wall amidase (CWA) and one cell-wall glucosamidase (CWG) domain separated by anchoring domains [11,13,14]. They are synthesized as propeptides and cleaved by an extracellular protease to generate two extracellular enzymes, namely a 51 kDa endo-β-*N*-acetylglucosaminidase (GHF-73) and a 62 kDa *N*-acetylmuramoyl-l-alanine amidase (NALAA-2), which are independently involved in the partitioning of daughter cells after cell division [15]. Atl was also shown to be involved in virulence by its adhesive properties and to elicit protective immunity against *S. aureus* and *S. epidermidis* [15,16] and in the internalization of *S. lugdunensis* by eukaryotic cells [17]. In *S. aureus*, Sle1 composed of three anchoring LysM domains and a C-terminal CHAP (cysteine, histidine-dependent amidohydrolase/peptidase) domain is also required for cell separation [18,19]. The CHAP domain is potentially involved in two different activities: as a peptidase, it cleaves between D-alanine and the first glycine of the pentaglycine cross-bridge and it can also act as an amidase by cleaving the chemical bond between MurNAc and L-alanine at the N-terminal of the stem peptide [20,21]. In *S. epidermidis* and *S. aureus*, Sle1, was essentially characterized for its adhesive properties to extracellular matrix proteins and further named Aae (autolysin/adhesin in *S. epidermidis*) and Aaa (autolysin/adhesin in *S. epidermidis*), respectively [6,22]. The murein hydrolase LytN harbors one LysM (instead of three as in SleI) and one CHAP domain, and promotes peptidoglycan separation and completion of the *S. aureus* cell cycle [23]. LytH, a l-Ala–d-Glu endopeptidase, identified in *S. aureus*, is involved in methicillin resistance [24]. 

Although these CWHs may be potentially promising staphylococcal lethal weapons, their lytic activity remains to be explored. This observation prompted us to inventory the CWHs of *S. epidermis* and explore their lytic potential. Here, we identify 16 hydrolases in the *S. epidermidis* RP62A strain, provide evidence of the activity of 11 representatives of the three catalytic domains and demonstrate the high potential of the Sle1 hydrolase in lysis of staphylococci.

## 2. Materials and Methods 

### 2.1. Bacterial Strains and Culture Conditions

Different strains of *Staphylococcus* belonging to nine species were used: 11 *Staphylococcus epidermidis* (RP62A, ATCC 12228, S06-011, S06-013, S06-022, S04-028, S04-036, S04-038, S04-056, S04-057, S04-058) isolated from medical samples [25,26], *Staphylococcus xylosus* C2a [27], *Staphylococcus aureus* (MW2, UAMS-1, Coch, SA113, S30) isolated from medical samples [25,28,29], *Staphylococcus simulans* DSM20273, *Staphylococcus saprophyticus* CIP 76.125^T^, *Staphylococcus hominis* CIP 81.57^T^, *Staphylococcus haemolyticus* CIP 81.56^T^, *Staphylococcus hyicus* DSM 20459^T^, and *Staphylococcus sciuri* CIP 81.62^T^. One strain of *Micrococcus aurantiacus* ATCC 11731 was also used. They were grown at 37 °C in Tryptic Soy Broth (TSB, Sigma-Aldrich, St. Louis, MO, USA) with orbital shaking (150 rpm) or on Tryptic Soy Agar plates (TSA, Sigma-Aldrich).

*Escherichia coli* strain TOP10 (Invitrogen, Carlsbad, CA, USA) was used as the cloning host for propagation of expression vector and used for protein expression. It was grown at 37 °C in lysogeny broth (LB) with orbital shaking (200 rpm) or on agar medium supplemented with ampicillin (100 µg/mL) when appropriate. Protein expression was induced by adding L-arabinose (0.2% *w*/*v*) (Sigma-Aldrich).

### 2.2. Proteogenomic Identification of CWHs

Considering the three classes of CWHs, the different conserved catalytic domains corresponding to CWAs, CWGs, and CWPs, as recently reviewed [10], were searched in *S. epidermidis* RP62A following a proteogenomic approach. These conserved domains were identified in proteins encoded in the genome of *S. epidermidis* RP62A following searches against InterPro (IPR) [30], which is composed of different member databases, such as Pfam [31], SMART [32], or the Conserved Domain database (CD) [33] where the identification of a conserved motif is based on a probabilistic match against HMM (hidden Markov model) or even PSSM (position-specific scoring matrix) profiles, which are more effective, relevant and robust than a percentage of identity or similarity against regular expressions [34].

### 2.3. Cloning and Expression of Hydrolases

DNA from *S. epidermidis* RP62A was used as a template to amplify the CDS encoding 11 different hydrolases. The primers used for PCR are listed in Table S1 and were synthesized by Eurofins Genomics. Amplifications were performed using Phusion High-Fidelity DNA polymerase (New England Biolabs) and the amplicons were then gel-purified using the QIAquick gel extraction kit (Qiagen, Hilden, Germany). PCR fragments were digested by *Kpn*I /*Eco*RI or *Pst*I /*Kpn*I and cloned in-frame downstream of the hexa-His box sequence in the pBAD/His B vector (Invitrogen) precut with the same restriction enzymes using T4 DNA Ligase (Roche, Basel, Switzerland). Plasmid constructions were transformed into *E. coli* TOP10 competent cells prior to selection on LB agar supplemented with ampicillin. *E. coli* TOP10 competent cells were also transformed with the empty pBAD/His B vector plasmid as a control. DNA sequencing was carried out to confirm the correct nucleotide sequence of the constructs using forward priming site and reverse priming site primers (Invitrogen, pBAD-F and pBAD-R, Table S1) and the Mix2Seq Kit (Eurofins Genomics).

Overnight precultures of *E. coli* recombinant strains were diluted 1:100 and grown in 100 mL of LB supplemented with ampicillin until OD_600 nm_ = 0.6. At this point, protein expression from pBAD was induced by adding L-arabinose and the cultures were incubated for a further 4 h at 37 °C and 20 h at 20 °C. Cells were harvested by centrifugation, washed in PBS (0.01 M, pH 8) and stored at −20 °C. They were suspended in 50 mM NaH_2_PO_4_, 300 mM NaCl buffer (pH 8) and disrupted with glass microbeads (400 mg per mL) using FastPrep (20 s twice at 6 m·s^−1^). Crude protein lysates were collected in the supernatant after elimination of unbroken cells and cellular debris by centrifugation (g, 45 min, 4 °C). The crude protein extracts and the control extract (i.e., the crude protein lysate from cells containing pBAD without insert) were then filtered through a 0.45 μm membrane filter and were frozen at −20 °C.

### 2.4. Western Blot Analysis

Protein concentrations were determined using the Bradford method [35]. The crude protein extracts were separated by SDS-PAGE after dilution in Laemmli buffer and heating to 95 °C for 5 min. Equal amounts of each extract were loaded per lane using Mini-PROTEAN TGX Precast Gels, 4%–15% (Bio-Rad). Precision Plus Protein Dual Color Standards (Bio-Rad) were used to enable molecular mass estimation as well as transfer control. Proteins resolved by SDS-PAGE were transferred to a PVDF membrane (iBlot^®^ 2 Transfer Stack) using iBlot^®^ 2 Dry Blotting system (Invitrogen). Membranes were blocked for 1 h using 10% skim milk in PBS and were probed with specific anti-His-Tag antibodies (dilution 1/1000 in PBS with 0.1% Tween and 2% skim milk, for 1.5 h at room temperature, Monoclonal Anti-polyhistidine-peroxidase antibody produced in mouse, Sigma-Aldrich). Finally, the SuperSignal West Dura kit (ThermoFisher, Waltham, MA, USA), a luminol-based enhanced chemiluminescent substrate, was used to detect peroxidase activity.

### 2.5. Cells Lysis Assay

Lysis assays of all crude protein extracts were performed. The *S. epidermidis* strain RP62a was grown in TSB with 1 M NaCl until OD_600 nm_ = 0.8. Cultures were centrifuged, washed and resuspended in Tris buffer (50 mM Tris-HCl, pH 7.0, Sigma-Aldrich) to OD_600 nm_ of 0.6. Subsequently, 100 µL of crude protein extract (1.6 ± 0.2 mg/mL) was added to 1 mL of cell suspension and placed in a cuvette covered with Parafilm. Cuvettes were incubated at 37 °C and lysis was followed at OD_600 nm_ at 0, 0.5, 1, 2, 3 and 4 h. The control extract was tested in the same conditions in each experiment. Lysis was expressed after subtraction of the control extract as a percentage of lysis from T0. The experiments were performed at least in triplicate.

The lytic activity of Sle1 crude extract was also tested against all *Staphylococcus* strains and the strain of *M. aurantiacus* as described above. It was studied at different temperatures of incubation (4, 10, 20, 25, 30, 37 °C) and different pHs (5.5, 7.0, 9.0) in Tris buffer adjusted with HCl or NaOH or in physiological saline solution (0.85%). It was also tested on biofilms of two *S. epidermidis* and *S. aureus* strains. Overnight precultures were adjusted at OD_600 nm_= 0.01 in TSB and 200 μL was loaded into the wells of a 96-well polystyrene microtiter plate prior to static incubation at 37 °C. After 24 h of incubation, the supernatant was removed and the adhered cells were washed. Crude protein extract of Sle1 (20 µL in 200 µL of Tris buffer) or control extract was then added. The microtiter plates were incubated for 4 h at 37 °C. The supernatant was removed and adhered cells were washed, fixed with alcohol, stained with 0.1% crystal violet, and treated with 33% acid acetic. The absorbance was read at 600 nm. Six biological replicates were carried out for each condition.

### 2.6. Phylogenetic Analysis of Sle1 Protein Sequence

A homology search was performed using the amino acid sequence of Sle1 from *S. epidermidis* RP62A as query following BLAST against UniProtKB [36,37]. The recovered protein sequences (*E*-value threshold 10^−10^) were aligned using T-Coffee [38]. The relatedness among protein sequences was studied using a Neighbor-Net phylogenetic network approach with SplitsTree v4.14.8 using the Hamming uncorrected-P distance [39]. The most robust branches were identified by bootstrap using a 90% threshold over 1000 pseudo-replicates.

## 3. Results

### 3.1. Selection of Cell-Wall Hydrolases in S. Epidermidis RP62A

**Figure 1:**
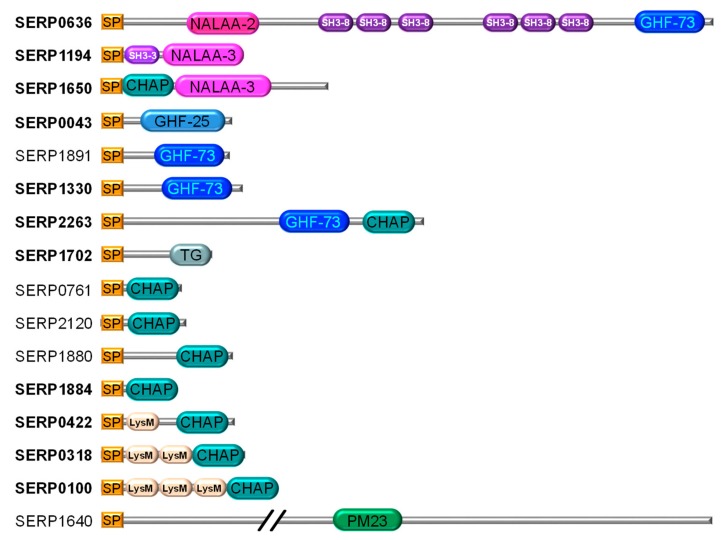
Schematic representation of cell-wall hydrolases (CWHs) identified in *Staphylococcus epidermidis* RP62A. SP: signal peptide, SH3, LysM: anchoring domain, NALAA: amidase domain, GHF: glycosidase domain, CHAP, PM23: peptidase domain, TG: transglycosylase. *S. epidermidis* RP62A locus names in bold were selected to study their lytic activity. SERP0636, SERP1194, SER0100 correspond to AtlE, LytH and Sle1, respectively.

The search in the UniProt database resulted in a list of 16 CWHs identified in S. epidermidis RP62A (Figure 1). They were classified according to their catalytic domains [10]. We identified CWHs with one catalytic domain, namely i) one CWA corresponding to N-acetylmuramoyl-L-alanine amidases (NALAA-3), ii) four CWGs including two *N*-acetylglucosaminidases (GHF-73), and one lysozyme (GHF-25) and a transglycosylase (TG), and iii) eight CWPs including seven Cysteine Histidine-dependent Amidohydrolases/Peptidases (CHAP) and one PM23. We also identified three bifunctional CWHs, one CWA/CWG (NALAA-2/GHF-73), one CWP/CWA (CHAP/NALAA-3) and one CWG/CWP (GHF-73/CHAP). Beside the catalytic domains, five of these CWHs have cell-wall binding domains such as SH3 (SH3-8, SH3-3) and LysM (Figure 1).

Eleven representative CWHs were selected to test their lytic activities against S. epidermidis RP62A (Figure 1). The corresponding genes without signal peptide sequence were cloned into expression vector. To ensure all the CWHs were expressed in E. coli, Western blot analyses of crude protein extracts were performed (Figure S1). All the CWHs were produced but the protein expression levels varied.

### 3.2. Lytic Activity of the 11 Hydrolases on Planktonic Cells of S. Epidermidis RP62A

**Figure 2:**
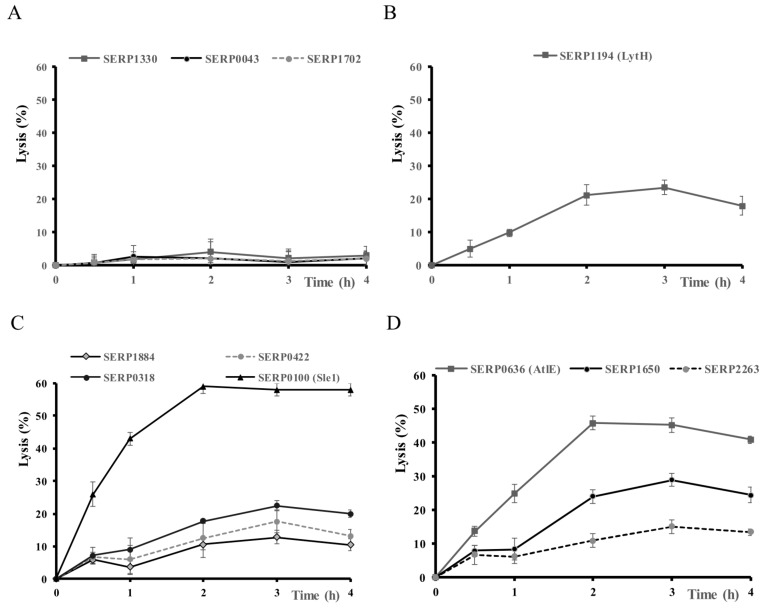
Lytic activity at pH 7.0 and 37 °C of the cell-wall glycosidase (**A**), amidase (**B**), peptidase (**C**) and bifunctional hydrolase (**D**) extracts on *Staphylococcus epidermidis* RP62A. Cell lysis was monitored at OD_600 nm_ over time, the control extract was subtracted and lysis was expressed as a percentage from T0.

The lytic activity of the 11 hydrolases was studied as crude protein extracts (1.6 ± 0.2 mg/mL) against S. epidermidis RP62A. The results are summarized according to their catalytic domains in Figure 2. Overall, the CWGs were not able to lyse S. epidermidis RP62A, while all the other CWHs could (Figure 2). Indeed, the two CWGs (SERP0043, SERP1330) and the transglycosylase (SERP1702) with three different catalytic domains (GHF-25, GHF-73, transglycosylase) and no anchoring domain (Figure 1) did not have any lysis activity (Figure 2A). The CWA SER1194, with a NALAA type 3 catalytic domain and SH3-3 anchoring in the N-terminal region (Figure 1), caused 20% of lysis (Figure 2 B). The four CWPs had lytic activities with three showing activity ranging from 13% to 25% while SRP0100 (Sle1) had a strong lytic activity with 59% of lysis after 3 h (Figure 2C) despite an apparent low production (Figure S1). The three CWPs SERP0422, SERP0318 and Sle1 have a CHAP catalytic domain but differ in their number of LysM anchoring domains (Figure 1). Sle1 had the highest number of LysM and was the most effective. The three bifunctional CWHs, although produced at the same level (Figure S1), lysed S. epidermidis with different efficiencies (Figure 2D). SERP0636 (AtlE) composed of two catalytic domains, NALAA-2/GHF-73, and six SH3-8 anchor domains (Figure 1), was the most active with 45% lysis (Figure 2D). The bi-functional enzymes, SERP1650 with CHAP/NALAA-3 and SERP2263 with GHF-73/CHAP catalytic domains, caused 29% and 15% of lysis, respectively (Figure 2D).

From these results, Sle1 appears as a promising CWH at least against *S. epidermidis* RP62A and was selected further characterization.

### 3.3. Diversity of Sle1

**Figure 3:**
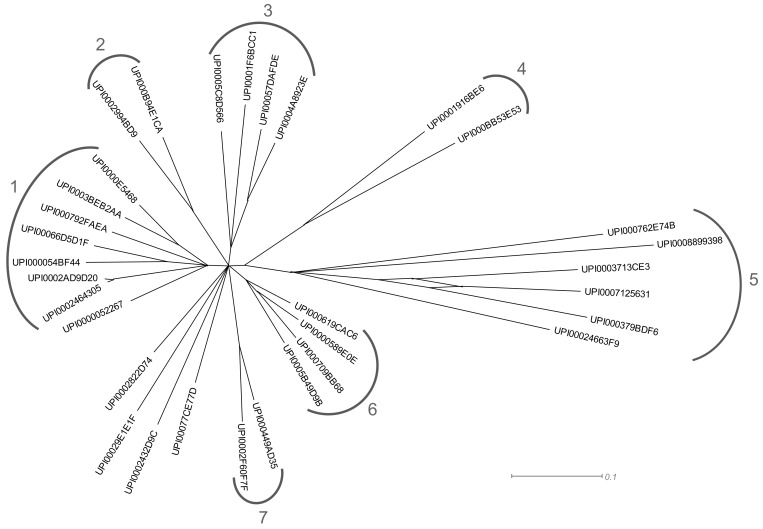
Phylogenetic tree based on Sle1 proteins (Table S2). The tree was constructed via SplitsTree4 by neighbor joining.

Sle1 is composed of three LysM anchoring domains and a catalytic CHAP domain (Figure 1). In order to position Sle1 from *S. epidermidis* RP62A in the Sle1 family, a phylogenetic analysis was carried out (Table S2). Following sequence alignment of Sle1 homologs, the phylogenetic tree revealed seven clusters and four deeply rooted branches (Figure 3). Cluster 1 includes Sle1 from *S. epidermidis* RP62A and constitues the largest group comprising staphylococcal Sle1 homologs such as *S. epidermidis-S. capitis*, *S. haemolyticus-S. hominis*, *S. warneri-S. pasteuri* clusters. Note that some Sle1 homologs found in some Gram-negative bacteria were also identified within this cluster (Table S2). With the exception of clusters 4 and 5, all other clusters also include Sle1 homologs essentially found within the genus *Staphylococcus*. It is of note that clusters 3 and 6 essentially comprised Sle1 homogs found in *S. hyicus-intermedius* and in *S. saprophyticus* species groups, respectively. Cluster 4 comprises Sle1 homogs from *Macrococcus* whereas cluster 5 includes homologs found in bacteria belonging to the *Lactobacillales* order and was the most distant.

### 3.4. Sle1 Active against Different Species of Staphylococcus

**Figure 4:**
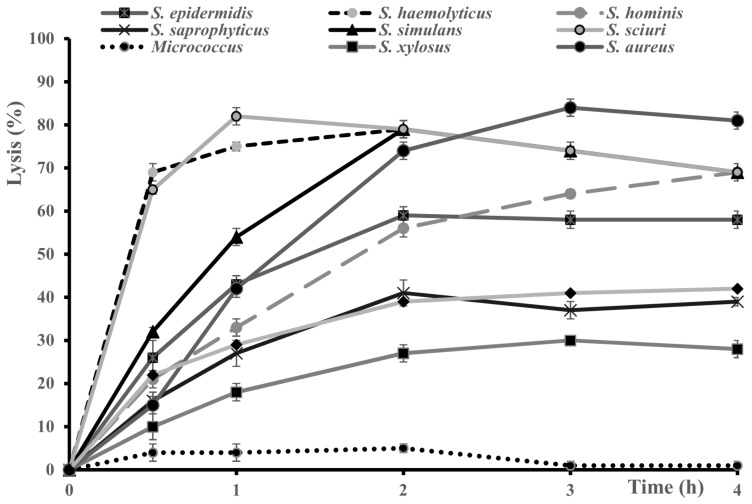
Lytic activity of Sle1 crude protein extract at pH 7.0 and 37 °C on several species of *Staphylococcus* and on *Micrococcus aurantiacus* (*S. epidermidis* RP62A, *S. haemolyticus* CIP 81.56^T^, *S. hominis* CIP 81.57^T^, *S. saprophyticus* CIP 76.125^T^, *S. simulans* DSM20273, *S. sciuri* CIP 81.62^T^*, S. xylosus* C2a, *S. aureus* UAMS-1, *S. hyicus* DSM 20459^T^, *M. aurantiacus* ATCC 11731). Cells lysis was monitored at OD_600 nm_ over time, the control extract was subtracted and lysis was expressed as a percentage from T0.

The lytic activity of Sle1 from *S. epidermidis* RP62a was further tested on eight species of staphylococci as well as *M. aurantiacus* (Figure 4). Lysis was observed for all staphylococci but not for the *Micrococcus* strain. The percentages of lysis were quite variable and ranged from 30% (against *S. xylosus*) to about 80% (against *S. sciuri, S. aureus, S. simulans*) after 2 h. Against *S. sciuri* (69%) and *S. haemolyticus* (65%) lysis occurred very quickly (within 30 min).

**Figure 5:**
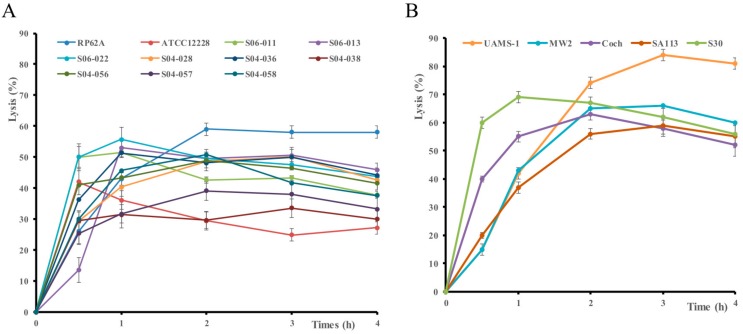
Lytic activity of Sle1 extract at pH 7.0 and 37 °C on (**A**) eleven strains of *Staphylococcus epidermidis* and (**B**) five strains of *Staphylococcus aureus.* Cell lysis was monitored at OD_600 nm_ over time, the control extract was subtracted and lysis was expressed as a percentage from T0.

Sle1 was also able to lyse 11 different strains of *S. epidermidis*, but with various efficacies depending on the strains (Figure 5A). The highest activity (59%) was noted against the strain RP62A and the lowest (30%) for S04-038. Similarly, four strains of *S. aureus* were studied and lysis reached a high percentage for the strain UAMS-1 (80%) and a lower one for the strain SA113 (59%). The S30 strain was really sensitive since 60% of the lysis was observed after 0.5 h incubation (Figure 5 B). The observed lysis was higher for the strains of *S. aureus* than for the strains of *S. epidermidis* (Figure 5). A mixture of *S. epidermidis* RP62A and *S. aureus* UAMS-1 in a 1:1 ratio was incubated with Sle1 extract. Sixty-one percent lysis was assayed for the mixture after 3 h of incubation (data not shown). In parallel, enumeration was carried out and at T0 was 8.3 log CFU/mL for the three populations (*S. epidermidis* RP62A, *S. aureus* UAMS-1, and the mixture of both) and a reduction of 3.0, 2.4 and 2.6 log CFU/mL was measured after 3 h of incubation with Sle1 extract for *S. aureus* UAMS-1, *S. epidermidis* RP62A and the mixture, respectively.

### 3.5. Sle1 Active in Different Environnemental Conditions

The lytic activity of Sle1 crude protein extract was compared to that of the CHAP domain alone. For this purpose, this domain was cloned into expression vector and expressed in *E. coli* as described above for the hydrolases. The CHAP crude protein extract had no lytic activity up to 2 h and a weak lytic activity between 15% and 10% after 4 h of incubation against *S. epidermidis* RP62A and *S. aureus* UAMS-1, respectively (data not shown).

**Figure 6:**
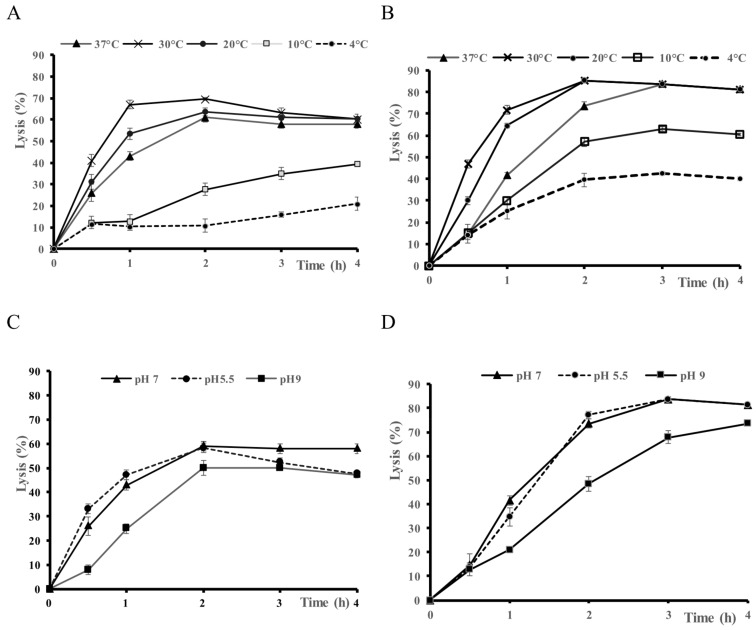
Lytic activity of Sle1 extract at pH 7.0 and different temperatures on (**A**) *Staphylococcus epidermidis* RP62A and (**B**) *Staphylococcus aureus* UAMS-1 and at 37 °C and different pHs on (**C**) *S. epidermidis* RP62A and (**D**) *S. aureus* UAMS-1. Cell lysis was monitored at OD_600 nm_ over time, the control extract was subtracted and lysis was expressed as a percentage from T0.

The lytic activity of Sle1 was assayed at different temperatures and pHs against *S. epidermidis* RP62A and *S. aureus* UAMS-1 (Figure 6). Lysis was maximal at 30 °C, and followed by 20 °C and then 37 °C. Lysis was still measured at 10 and 4 °C (Figure 6A,B). The lytic activity at 37 °C was close at pH 5.5 and 7.0 while it was lower at pH 9.0 (Figure 6C,D).

The Sle1 enzyme extract kept its activity after one night at 4 °C or at room temperature. It had the same activity in Tris buffer and in physiological saline solution (data not shown).

**Figure 7:**
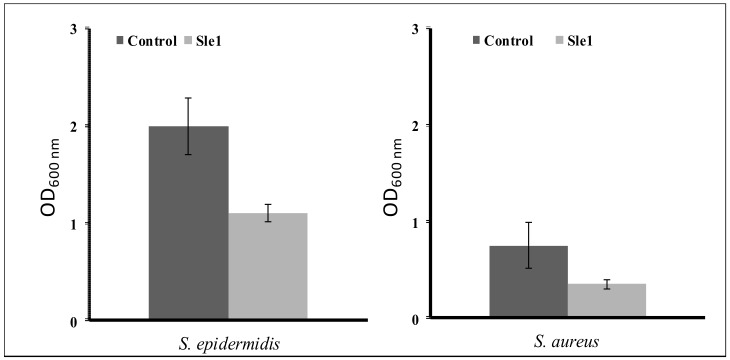
Effect of Sle1 extract on biofilm of *Staphylococcus epidermidis* ATCC 12228 and *Staphylococcus aureus* MW2.

The activity of Sle1 was also tested on biofilms of the *S. epidermidis* ATCC 12228 and *S. aureus* MW2 strains, these strains forming more biofilm than the strains RP62A and UAMS-1, respectively, in our experimental conditions (data not shown). The Sle1 extract was able to detach adhered cells of *S. epidermidis* and *S. aureus*, by 45% and 60% less, respectively, than the control extract (Figure 7).

## 4. Discussion

Of the 16 CWHs identified in the strain *S. epidermidis* RP62A, 14 have not yet been reported in *S. epidermidis*, while two, Atl and Sle1, have already been identified in *S. epidermidis* and mainly characterized for their adhesive properties [6,40]. LytH, which we identified in *S. epidermidis*, has already been studied in *S. aureus* just like Sle1 [41]. Moreover, SERP1330, SER1891 and SRP2263 are homologous to LytX, LytY, and LytZ from *S. aureus*, respectively [23]. In our study, the CHAP domain was found in nine CWHs out of the 16 identified in *S. epidermidis* RP62A. This domain is found in a wide range of protein architectures [20]. It was also identified in 11 CWHs in 12 strains of *S. aureus* and in 44 staphylococcal phages [42]. It is associated with the CWA or CWG domain in 2 CWHs of *S. epidermidis* RP62A (Figure 1) and five in *S. aureus* [42].

A representative set of the three categories of CWHs, namely CWGs, CWAs and CWPs, was tested for their lytic activity against living cells of *S. epidermidis* RP62A. The two CWGs and the transglycosylase did not show any lytic activities (Figure 2). The CWG SERP0043 belongs to the glycoside hydrolase family GH-25. The structure resolution of the GHF-25 protein LytC, from *Streptococcus pneumoniae*, revealed that this enzyme was only able to hydrolyze non-cross-linked peptidoglycan chains [43]. The other CWG SERP1330 belongs to the GH-73 family. The structure of the GH-73 from *Listeria monocytogenes* revealed that it is inactive when newly secreted but activated by proteolytic cleavage [44]. In *E. coli*, transglycosylase and transpeptidase (peptidoglycan synthases) formed enzymatic complexes with CWHs and are involved in cell division and cell wall rearrangement [45,46,47]. The CWA LytH of *S. epidermidis* RP62A, composed of an NALAA-3 catalytic domain and an SH3 anchoring one, lysed living cells of *S. epidermidis* while LytH from *S. aureus* was not able to lyse *S. aureus* living cells [41]. In *S. aureus*, its absence was correlated with methicillin resistance [24].

The three bifunctional enzymes we studied, AtlE (CWA/CWG), SERP1650 (CWP/CWA) and SERP2263 (CWG/CWP) lysed living cells of *S. epidermidis*. Atl was the most efficient and is one of the most studied CWHs in staphylococci. The domain arrangement of the Atl precursor protein is conserved among the staphylococci [15]. In *S. aureus* and *S. epidermidis*, Atl is post-translocationally processed between the propeptide and amidase domain and between the repeat anchoring domains [15]. In the *S. epidermidis* strain RP62A, it could be between the fourth and fifth SH3-8 (Figure 1) to free amidase SH3-8 (4 repeats) and glycosidase SH3-8 (2 repeats). The Western blot analysis of the Atl crude protein extract revealed two bands that could correspond to the native enzyme and the amidase. The lytic activity of Atl has been little explored, whereas its involvement in cell division, adhesive properties and biofilm formation and its protective effect against immunity have been widely studied [13,14,15,16,17,48,49].

The four CWPs studied (Figure 2) lysed living cells of *S. epidermidis* RP62A. They are all composed of a CHAP catalytic domain and variable LysM anchoring domains. The lytic activity increased with the number of LysM, with Sle1 with three LysM being the most active. Due to the high lytic activity of Sle1 against *S. epidermidis* RP62A, we focused on this CWH. Sle1, formerly named Aae for *S. epidermidis* and Aaa for *S. aureus*, has only been reported in these two species [6,18,22,50].

Sle1 harbors a CHAP catalytic domain and three LysM domains. We first confirmed the role of LysM domains in lysis by showing that CHAP extract had a low activity compared to Sle1 extract. LysM domains of Sle1 of *S. aureus* direct murein hydrolases to the staphylococcal envelope [19]. The LysM domains bind to the repeating disaccharide of staphylococcal peptidoglycan. Sle1 of *S. aureus* requires at least two or three LysM domains to properly localize to the cell division septum [19]. The CHAP catalytic domain could act as a peptidase or an amidase [20]. In *S. aureus*, Sle1 was shown to act preferentially as an amidase and was involved in cell separation [18]. The CHAP catalytic domain of *S. aureus* LytN functions as both an amidase and a peptidase and promotes peptidoglycan separation [23]. In *S. epidermidis* and *S. aureus*, Sle1 has essentially been characterized for its adhesive properties to extracellular matrix proteins, but it also shows lytic activity, albeit only on heat-inactivated cells of *Staphylococcus carnosus* [6].

In this study, Sle1 homologs appeared to be distributed into seven distinct clusters and were identified in 24 staphylococcal species but also in *Macrococcus*, in bacteria from the *Lactobacillale* order and in Gram-positive and Gram-negative bacteria belonging to different genera. Within a given species, Sle1 homologs belonging to different clusters could be identified. The diversity of CHAP domains is apparent from phylogenetic analysis of 12 sequenced genomes of *S. aureus* and reveals three categories: i) proteins identified in all the genomes and with the CHAP domain at the C-terminal region, ii) proteins identified in three of the 12 genomes, encoded by chromosome and/or by plasmid and with the CHAP domain at the C-terminal region, and iii) proteins not identified in all genomes, with the CHAP domain at the N-terminal region prophage-encoded [42].

Our study demonstrated a broad spectrum of lysis against living cells of nine different species of staphylococci. Lysis was recorded against living cells of different strains of *S. epidermidis* and *S. aureus.* A large spectrum of lysis of staphylococcal species was also noted for the mutant CW hydrolase LysF1 (CHAP domain and SH3b binding domain) from the staphylococcal bacteriophage 812F1 [51]. Like Sle1, LysF1 exhibited different antibacterial activities. In LysF1, this difference could be attributed to the effect of the binding domain SH3b on the different peptidoglycans [51]. Engineered phage derived lytic enzymes such as LysF1 improved their potential as antimicrobials and open new possibilities to tackle bacterial infections [52]. Sle1 was active at different temperatures between 37 and 4 °C, at different pHs between 5.5 and 9 and at 145 mM NaCl. LysK from a staphylococcal phage harboring a CHAP domain had a strong activity between pH 6 and 9 and showed increasing activity from 150 mM to 400 mM NaCl [53]. Similarly, the hydrolase of staphylolytic Twort phage with a CHAP domain had a maximum activity at 300 mM NaCl [54]. The activities of *S. aureus* lysostaphin and LytM, two other peptidases that harbor PM23 as catalytic domain, depended differently on pH: they were marginally active at pH 6.0, very active at pH 7.0 to 9.0 for lysostaphin and pH 8.0 to 9.0 for LytM [55].

Sle1 of *S. epidermidis* RP62A was able to detach sessile cells of *S. epidermidis* and *S. aureus* from abiotic surfaces. To date, the role of CWHs in the disruption of biofilm was essentially documented for lysostaphin, which can eradicate *S. aureus* biofilms, at least when the strains are not lysostaphin-resistant, but also *S. epidermidis* biofilms if higher concentrations of enzyme are used [56].

## 5. Conclusions

*S. epidermidis* has several CWHs with different lytic potentials. One of them, Sle1, exhibited a broad spectrum of staphylococcal lysis. It was active at different temperatures, pHs, in saline solution, against planktonic and sessile cells of *S. epidermidis* and *S. aureus* strains. If further studies show the activity of Sle1 in in vivo models, it could be an alternative or a complementary therapy to antibiotic treatment for infections caused by staphylococci.

## Supplementary Materials

The following are available online at https://www.mdpi.com/2076-2607/7/11/559/s1, Figure S1: Western blot analysis of cell-wall hydrolase crude protein extracts, Table S1: Primers used in this study, Table S2: The amino acid sequences of Sle1were searched in the UniProt data base using the Sle1 sequence of *S. epidermidis* RP62A as template.
