# Supplementary material for: Cell-Wall Hydrolases as Antimicrobials against Staphylococcus Species: Focus on Sle1
Source: Microorganisms. 2019 Nov 12;7(11):559. doi: 10.3390/microorganisms7110559 (PMC6921076; doi:10.3390/microorganisms7110559)
Supplement: Supplementary file 1 [file microorganisms-07-00559-s001.zip › FIGURE S1.pdf]

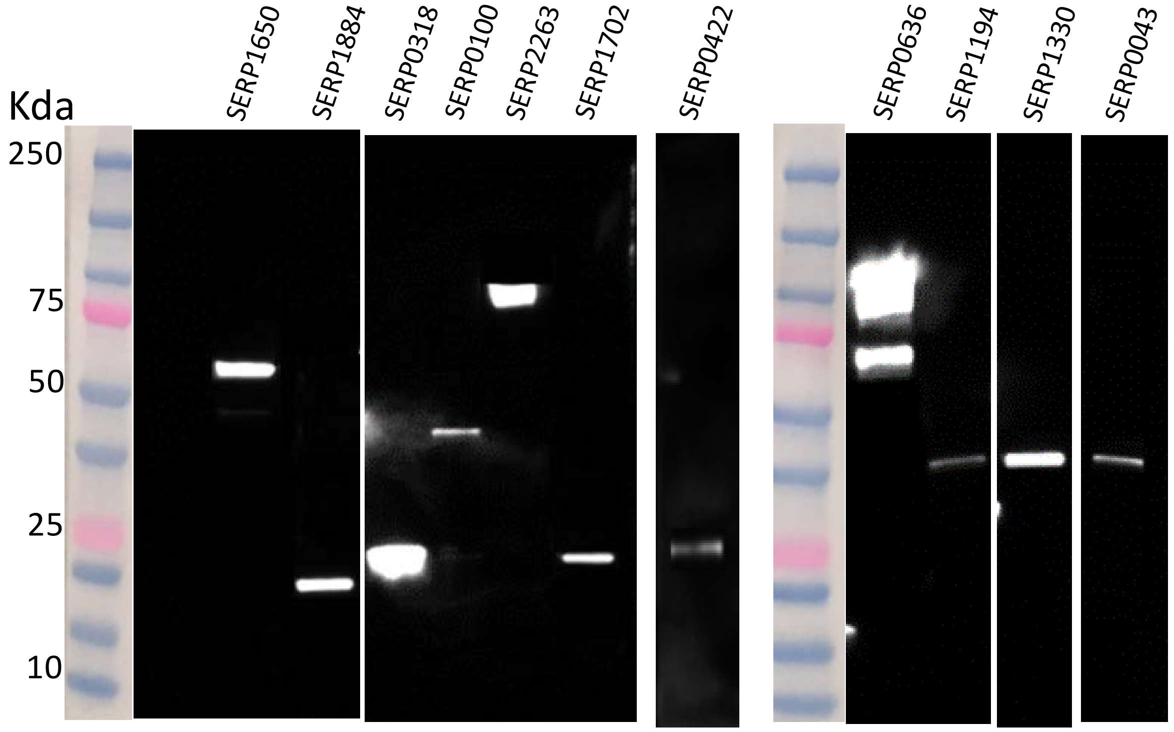

Figure S1: Western blot analysis of cell-wall hydrolases crude protein extracts from *E. coli* Top10 pBAD/His containing the respective inserts.
