# Supplementary material for: Cell-Wall Hydrolases as Antimicrobials against Staphylococcus Species: Focus on Sle1
Source: Microorganisms. 2019 Nov 12;7(11):559. doi: 10.3390/microorganisms7110559 (PMC6921076; doi:10.3390/microorganisms7110559)
Supplement: Supplementary file 1 [file microorganisms-07-00559-s001.zip › TableS1.pdf]

**Table S1:** Primers used in this study

| Primer name | Primer sequence (restriction site underlined) |
|---|---|
| pBAD-F | 5'-ATGCCATAGCATTTTTATCC-3' |
| pBAD-R | 5'-CCTGATACAGATTAAATCAG-3' |
| SERP0043F1 | 5'-AACTGCAGCTGGGTATGGCTATCAACAATAC-3' |
| SERP0043R1 | 5'-GAATTCGTCTTGGTTCACTTCATGAGGTACCCC-3' |
| SERP0100F1 (*sle1*) | 5'-AACTGCAGCTGCAACAACGCATACAGTAA-3' |
| SERP0100R1 (*sle1*) | 5'-CAAGTGAGAAATTACAAATTTATTCATTGAGGTACCCC-3' |
| SERP0318F1 | 5'-GGGGTACCGATGCTGAAGCTTCTACACAA-3' |
| SERP0318R1 | 5'-GTATCTTCATATGCATTTATCCACTGAGAATTCCG-3' |
| SERP0422F1 | 5'-GGGGTACCGCTAACGCACAAGAATCTGA-3' |
| SERP0422R1 | 5'-CCCTAATTCATATAATTACATTCACTGAGAATTCCG-3' |
| SERP0636F1 (*atlE*) | 5'-AACTGCAGCTGTATCTAGTCAAAAAAC-3' |
| SERP0636R1 (*atlE*) | 5'-GAAATTCCAACATACAAATGAGGTACCCC-3' |
| SERP1194F1 (*lytH*) | 5'-AACTGCAGCTGAAGATACAGGTCAAATTACT-3' |
| SERP1194R1 (*lytH*) | 5'-GGTTTAAAACAATATTTCTCGTCCTGAGGTACCCC-3' |
| SERP1330F1 | 5'-GGGGTACCATCTTCTTTTCAATGATTTCAGATC-3' |
| SERP1330R1 | 5'-TAAAGATGACGATAAACACCAAAAATGAGAATTCCG-3' |
| SERP1650F1 | 5'-AACTGCAGCTGTTGATCGTAGCTTCCATGC-3' |
| SERP1650R1 | 5'-GAATTATATGGTAAAATTAAATGGAAGTGAGGTACCCC-3' |
| SERP1702F1 | 5'-GGGGTACCGCAAGTGAGACTACAAATGTTG-3' |
| SERP1702R1 | 5'-CAGGTCATTGGGTAACTGCATGAGAATTCCG-3' |
| SERP1884F1 | 5'-GGGGTACCGCTGAAAATAACAATCAACAACAAA-3' |
| SERP1884R1 | 5'-TCATATAATTACATCCACCTGAATTGAGAATTCCG-3' |
| SERP2263F1 | 5'-GGGGTACCGAAGATTCATCTAATAAAAATACAAATT-3' |
| SERP2263R1 | 5'-CAAGATTTAGATTACATTAAAGGTAAATGAGAATTCCG-3' |
